# Supplementary material for: Thermal sensors improve wrist-worn position tracking
Source: NPJ Digit Med. 2019 Mar 14;2:15. doi: 10.1038/s41746-019-0092-2 (PMC6550265; doi:10.1038/s41746-019-0092-2)
Supplement: Supplementary file 1 — Supplementary Tables. [file 41746_2019_92_MOESM1_ESM.pdf]

Supplementary Table 1 | Discriminability distance distribution and p-values from Wilcoxon signed-rank tests for the Tingle *with no* thermal sensor data between the sampling distribution and original distance (median ± MAD)

Target pair	Discriminability distance: Shuffled labels	Discriminability distance: True labels	p-value	Effect size
**Mouth - Nose**	0.16 ± 0.07	2.11 ± 1.08	3.50e-07	3.37
**Mouth - Cheek**	0.17 ± 0.10	2.30 ± 0.44	3.57e-08	3.02
**Mouth - Eyebrow**	0.16 ± 0.04	1.67 ± 1.08	7.59e-08	2.71
**Mouth - Top-head**	0.15 ± 0.06	2.32 ± 0.66	4.16e-08	2.63
**Mouth - Back-head**	0.23 ± 0.11	2.59 ± 0.57	3.57e-08	1.40
**Nose - Cheek**	0.09 ± 0.08	0.49 ± 0.46	4.29e-06	1.71
**Nose - Eyebrow**	0.11 ± 0.06	0.85 ± 0.90	5.91e-06	2.41
**Nose - Top-head**	0.22 ± 0.22	2.19 ± 0.91	2.45e-07	1.38
**Nose - Back-head**	0.18 ± 0.13	2.12 ± 1.12	3.85e-08	2.00
**Cheek - Eyebrow**	0.10 ± 0.08	0.77 ± 0.81	1.83e-06	1.87
**Cheek - Top-head**	0.20 ± 0.16	2.18 ± 0.67	1.37e-07	2.37
**Cheek - Back-head**	0.22 ± 0.21	1.63 ± 1.12	3.85e-08	1.71
**Eyebrow - Top-head**	0.19 ± 0.14	1.91 ± 1.01	5.63e-08	3.26
**Eyebrow - Back-head**	0.22 ± 0.19	2.33 ± 0.78	3.57e-08	1.78
**Top-head - Back-head**	0.22 ± 0.13	2.11 ± 0.90	1.37e-07	1.94

Supplementary Table 2 | Discriminability distance distribution and p-values from Wilcoxon signed-rank tests for the Tingle *with* thermal sensor data between the sampling distribution and original distance (median ± MAD)

Target pair	Discriminability distance: Shuffled labels	Discriminability distance: True labels	p-value	Effect size
**Mouth - Nose**	0.62 ± 0.21	3.06 ± 1.04	6.07e-08	1.91
**Mouth - Cheek**	0.71 ± 0.20	3.07 ± 0.94	4.84e-08	1.62
**Mouth - Eyebrow**	0.62 ± 0.20	2.61 ± 0.75	8.18e-08	2.04
**Mouth - Top-head**	0.65 ± 0.11	2.94 ± 0.48	3.57e-08	1.51

**Mouth - Back-head**	0.76 ± 0.23	3.31 ± 0.47	3.57e-08	1.35
**Nose - Cheek**	0.52 ± 0.18	1.25 ± 0.84	3.76e-07	1.62
**Nose - Eyebrow**	0.62 ± 0.38	2.17 ± 0.84	7.56e-07	1.43
**Nose - Top-head**	1.49 ± 1.14	3.27 ± 0.91	9.29e-07	1.26
**Nose - Back-head**	0.82 ± 0.41	3.32 ± 0.66	8.68e-07	1.28
**Cheek - Eyebrow**	0.63 ± 0.32	2.23 ± 1.32	2.09e-06	1.75
**Cheek - Top-head**	1.05 ± 0.65	3.22 ± 0.96	9.18e-06	1.22
**Cheek - Back-head**	0.82 ± 0.40	3.01 ± 0.72	6.54e-08	1.31
**Eyebrow - Top-head**	0.77 ± 0.38	2.71 ± 0.91	3.31e-06	1.32
**Eyebrow - Back-head**	0.84 ± 0.33	3.01 ± 0.77	3.57e-08	1.51
**Top-head - Back-head**	0.89 ± 0.40	2.88 ± 0.50	5.22e-08	1.29

Supplementary Table 3 | AUROC value distribution (for individual and general classifiers) and p-values from Wilcoxon signed-rank tests for the Tingle *with no* thermal sensor data and *with* thermal sensor data (median ± MAD)

	**Mouth**	**Nose**	**Cheek**	**Eyebrow**	**Top-head**	**Back-head**
**No thermal**	0.86 ± 0.13	0.69 ± 0.26	0.60 ± 0.15	0.74 ± 0.36	0.91 ± 0.12	0.73 ± 0.33
**Yes thermal**	0.99 ± 0.01	0.96 ± 0.07	0.93 ± 0.09	0.96 ± 0.06	0.98 ± 0.06	1.00 ± 0.00
**p-value**	3.20e-07	1.14e-07	6.15e-08	2.48e-07	3.65e-07	7.25e-08
**Effect size**	1.27	1.37	1.93	1.66	0.94	3.49
**General**	0.89 ± 0.13	0.83 ± 0.13	0.75 ± 0.15	0.84 ± 0.07	0.89 ± 0.10	0.94 ± 0.08
